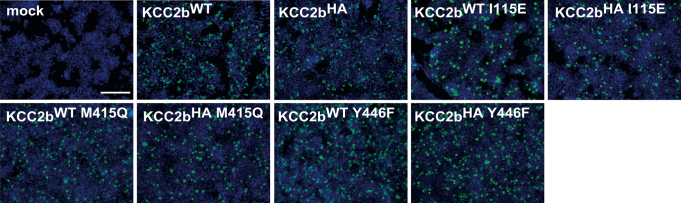# Both chloride-binding sites are required for KCC2-mediated transport

**DOI:** 10.1016/j.jbc.2023.105190

**Published:** 2023-08-23

**Authors:** Lisa Becker, Jens Hausmann, Anna-Maria Hartmann

**Affiliations:** 1 Division of Neurogenetics, School of Medicine and Health Sciences, Carl von Ossietzky University Oldenburg, Oldenburg, Germany; 2 Division of Anatomy, School of Medicine and Health Sciences, Carl von Ossietzky University Oldenburg, Oldenburg, Germany; 3 Research Center for Neurosensory Sciences, Carl von Ossietzky University Oldenburg, Oldenburg, Germany

**Keywords:** structure, site-directed mutagenesis, protein conformation, cation chloride cotransporter, cell culture, chloride-binding site

## Abstract

The K^+^–Cl^−^ cotransporter 2 (KCC2) plays an important role in inhibitory neurotransmission, and its impairment is associated with neurological and psychiatric disorders, including epilepsy, schizophrenia, and autism. Although KCCs transport K^+^ and Cl^−^ in a 1:1 stoichiometry, two Cl^−^ coordination sites were indicated *via* cryo-EM. In a comprehensive analysis, we analyzed the consequences of point mutations of residues coordinating Cl^−^ in Cl_1_ and Cl_2_. Individual mutations of residues in Cl_1_ and Cl_2_ reduce or abolish KCC2^WT^ function, indicating a crucial role of both Cl^−^ coordination sites for KCC2 function. Structural changes in the extracellular loop 2 by inserting a 3xHA tag switches the K^+^ coordination site to another position. To investigate, whether the extension of the extracellular loop 2 with the 3xHA tag also affects the coordination of the two Cl^−^ coordination sites, we carried out the analogous experiments for both Cl^−^ coordinating sites in the KCC2^HA^ construct. These analyses showed that most of the individual mutation of residues in Cl_1_ and Cl_2_ in the KCC2^HA^ construct reduces or abolishes KCC2 function, indicating that the coordination of Cl^−^ remains at the same position. However, the coupling of K^+^ and Cl^−^ in Cl_1_ is still apparent in the KCC2^HA^ construct, indicating a mutual dependence of both ions. In addition, the coordination residue Tyr^569^ in Cl_2_ shifted in KCC2^HA^. Thus, conformational changes in the extracellular domain affect K^+^ and Cl^−^-binding sites. However, the effect on the Cl^−^-binding sites is subtler.

The amino acid/polyamine/organocation superfamily comprises the second largest superfamily of secondary carriers (1), initially described by Saier *et al.* (2). Central to the superfamily members is the LeuT-fold (3, 4, 5), which is mainly characterized by a conserved five-helix transmembrane inverted repeat motif (5 + 5 topology). This fold is highly conserved within the amino acid/polyamine/organocation superfamily. A family within this superfamily is the cation chloride cotransporter (CCC) family (3, 4, 5). The CCCs consist of secondary active membrane transporters that mediate the symport of cations (Na^+^ and K^+^) coupled with chloride (Cl^−^) (6). Gene duplication events at the base of archaeans and eukaryotes led to the diversification and neofunctionalization of the paralogous CCC subfamilies: the Na^+^ K^+^ 2Cl^−^ cotransporter (NKCC1+2) and Na^+^ Cl^−^ cotransporter (NCC), the K^+^–Cl^−^ cotransporter (KCC1–4), the polyamine transporter CCC9, and the CCC-interacting protein CIP1 (6, 7). NKCCs and NCCs mediate the inward-directed transport of Na^+^, (K^+^), and Cl^−^, and KCCs mediate the outward-directed transport of K^+^ and Cl^−^ (6). Both use Na^+^ and K^+^ gradients, which are built up by the Na^+^/K^+^ ATPase, to transport Cl^−^ against its electrochemical gradient (6). CCC family members play an essential physiological role in transepithelial ion reabsorption and secretion, cell volume regulation, and Cl^−^ homeostasis (6, 8, 9, 10).

Structurally, CCCs consist of 12 transmembrane helices (TMs), flanked by the intracellular N and C terminus, as well as a large extracellular domain (ECD) (6, 7, 11, 12, 13). The ECD in KCCs is situated between TMs 5 + 6 and in N(K)CCs between TM 7 + 8 (6, 11, 12, 14, 15). The termini are important for trafficking, isotonic activity, dimerization, and regulation *via* post-translational modifications (13, 16, 17, 18, 19, 20, 21, 22). The N-terminal domain of KCCs has also an autoinhibitory function, since this domain seals the entrance of the transporter in the inward–open configuration, and thus, ion translocation cannot occur (21, 23, 24, 25, 26). 3D reconstruction of CCCs from single-particle cryo-EM demonstrated the inverted repeat of TMs 1 to 5 and TMs 6 to 10 (15, 20, 21, 23, 27, 28, 29, 30, 31, 32, 33, 34, 35, 36). Accordingly, TMs 11 + 12 are located outside this structural element (36, 37, 38, 39, 40).

Within the CCC family, the KCC2 is of particular interest. It is the major Cl^−^ extrusion transporter in most adult inhibitory neurons of the central nervous system where it lowers [Cl^−^]_i_ (9, 22, 41). Its action enables fast hyperpolarizing postsynaptic inhibition because of Cl^−^ influx mediated by the binding of the inhibitory neurotransmitters gamma-aminobutyric acid and glycine to their ionic gamma-aminobutyric acid type A and glycine receptors (9, 41). Mice with disruption of the gene *SLC12A5*, which encodes both KCC2 splice variants (KCC2a and KCC2b), die shortly after birth because of motor deficits (42, 43). Dysfunction of KCC2 is associated with several neurological and psychiatric disorders, including epilepsy, neuropathic pain, spasticity, ischemic insults, brain trauma, schizophrenia, and autism (44, 45, 46, 47, 48, 49, 50, 51, 52, 53, 54). These severe consequences raised pharmacological interest in the generation of small-molecule KCC2 potentiators that enhance its activity (55, 56, 57). This requires a thorough understanding of the structure and functioning of KCC2.

An important prerequisite is the knowledge on the residues that mediate the ion translocation. The amino acid residues coordinating Na^+^, K^+^, and Cl^−^ are highly conserved in KCC1–4, NKCC1, and NCC (21, 24, 27, 28, 29, 34, 35, 36), suggesting a similar mechanism of Cl^−^ coupling and K^+^ transport (27, 58). Functional analyses in KCC1 (Tyr in TM3), KCC2 (Asn and Ile in TM1, Tyr in TM3, and Pro and Thr in TM6), KCC3 (Tyr in TM3 and Thr in TM6), KCC4 (Asn in TM1 and Tyr in TM3), and NKCC1 (Tyr in TM3) confirmed this view (27, 28, 29, 59).

Cryo-EM data suggest two Cl^−^-binding sites in NKCCs, in agreement with their stoichiometric transport of 1 Na^+^:1 K^+^:2 Cl (28, 34). Unexpectedly, cryo-EM structures of KCC1, KCC3, and KCC4 revealed densities for two Cl^−^--binding sites, despite their transport of K^+^ and Cl^−^ in a stoichiometric 1:1 ratio (21, 24, 29). These two Cl^−^ coordinating sites are highly conserved in KCCs and N(K)CCs (Fig. 1) (20, 21, 23, 27, 28, 29, 58). The first coordinating site (Cl_1_) harbors conserved residues mainly in TM1 (Gly^113^, Val^114^, and Ile^115^, annotation according to murine KCC2b, Figs. 1 and 2*A*) and was revealed in cryo-EM structures of NKCC1, NCC, KCC1, KCC3, and KCC4 (20, 23, 27, 28, 29, 35, 58). Molecular dynamics (MD) simulation in addition proposed the presence of the Cl_1_ site in KCC2 (20). The second Cl^−^ coordination site (Cl_2_) is composed of main-chain interaction residues from TM6 (Gly^413^, Ile^414^, and Met^415^) and Tyr^569^ in TM10 (Fig. 2*A*) (20, 21, 27, 35, 58). These coordination sites were revealed in cryo-EM structures of NKCC1, NCC, and KCC1–KCC4 (21, 24, 27, 28, 29, 34, 35). Mutation of Tyr in TM10 (Cl_2_) reduces or abolishes the transport activity of KCC1, KCC3, KCC4, NKCC1, and NCC indicating that the Cl_2_ site is important for their function (27, 28, 29, 35).

**Figure 1:**
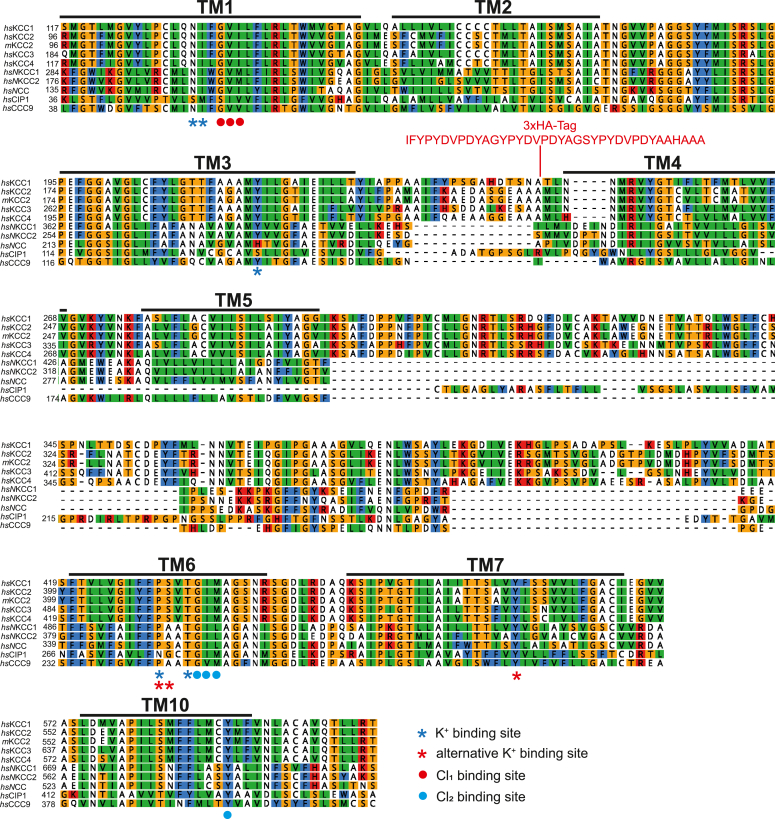
**Evolutionary conversation of K**^**+**^**and Cl**^**−**^**binding site.** Multialignment of TM1 to TM10 of mouse KCC2 (NM_020333.2), human KCC1 (NP_005063.1), KCC2 (NP_065759.1), KCC3 (NP_598408.1), KCC4 (NP_006589.2) and NKCC1 (NP_001037.1), NKCC2 (NP_001171761.1), NCC (NP_000330.2), CIP1 (NP_064631.2), and CCC9 (NP_078904.3) was generated using ClustalW in Geneious. The TMs, K^+^, and Cl^−^-binding sites were annotated according to the cryo-EM–based three-dimensional structure of *mm*KCC2 and *hs*NKCC1 (20). Cl^−^-binding sites in Cl_1_ located in TM1 (G, V, and I) are marked with *red dots*. Cl^−^-binding sites in Cl_2_ located in TM6 (G, I, and M) and TM10 (Y) are marked with *blue dots*. K^+^-binding sites located in TM1 (N and I), TM3 (Y), and TM6 (P and T) are marked with a *blue asterisk*. Alternative K^+^ sites located in TM6 (P and S) and TM7 (Y) are marked with a *red asterisk*. The insertion of the HA tag of KCC2b^HA^ at the end of EL2 between TM3 and TM4 is highlighted in *red*. HA, hemagglutinin; *hs*, *Homo sapiens*; KCC, K^+^–Cl^−^ cotransporter; *mm*, *Mus musculus*; TM, transmembrane helix.

**Figure 2:**
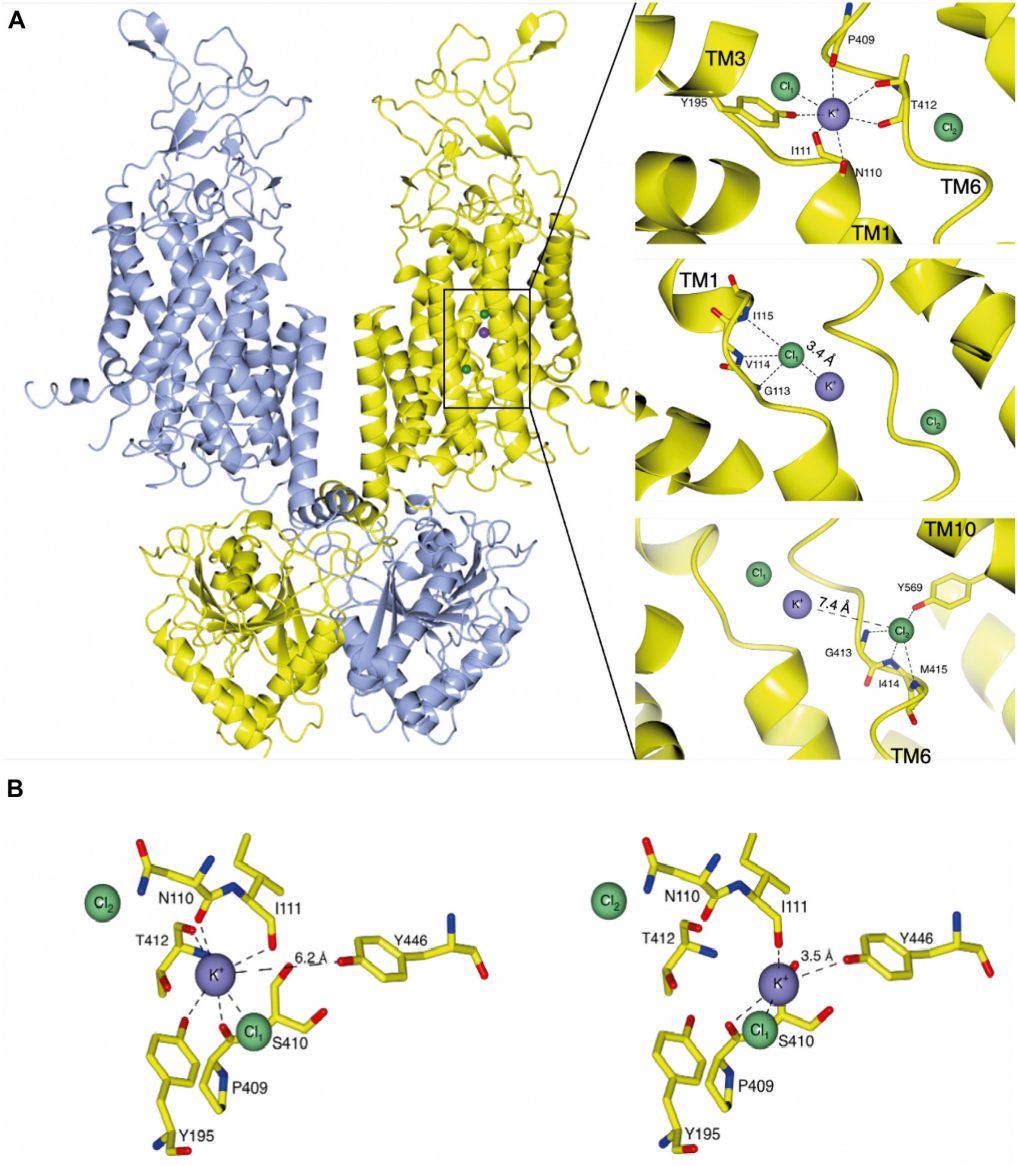
**Structural homology model of KCC2 based on PDB entry**7D99**.***A*, structural overview of the KCC4 dimer (PDB code: 7D99), the closest homolog of *mm*KCC2, colored by its homodimeric subunits in *yellow* and *blue*. Cl^−^ ions are presented as *green spheres*, and the K^+^ ion is shown as a *purple sphere*. *Right side upper*, zoom into the K^+^ coordination site. *Right side middle*, close-up of the first Cl^−^ ion-binding site (Cl_1_). *Right side down*, atoms of residues participating in the coordination are indicated by *dashed lines*. *B*, structural comparison between the K^+^-binding site as observed in 7D99 (*left*) and the suggested alternative binding site (*right*). Shown are residues that coordinate the K^+^ ion. The residue annotation is according to the *rn*KCC2b construct. KCC, K^+^–Cl^−^ cotransporter; *mm*, *Mus musculus*; PDB, Protein Data Bank; *rn*, *Rattus norvegicus*.

None of these Cl^−^-binding sites have been functionally investigated in KCC2, and more importantly, it is still not known whether both sites are required for transport. Therefore, we performed an extensive functional analysis of residues coordinating Cl^−^ in Cl_1_ and Cl_2_ sites. Our analyses revealed that both Cl^−^ coordinating sites are required for KCC2 function.

Recently, we showed that a 3xHA tag in KCC2 EL2 connecting TM3 and TM4 (KCC2^HA^) results in subtle conformational changes of the ECD and affects K^+^ ion binding (59). This conformational change likely switches the classical K^+^-binding site to an alternative K^+^-binding site consisting of Pro^409^ and Ser^410^ in TM6 and Tyr^446^ in TM7 (Fig. 2*B*) (59). Here, we used the KCC2^HA^ construct to analyze, whether the extension of the EL2 also leads to a shift of the Cl_1_ and Cl_2_ sites. Our data show that this conformational change affects ion coordination of both K^+^ and both Cl^−^, indicating a functional coupling of these sites.

## Results

### Cl^−^-binding sites in KCC2b

**Figure 3:**
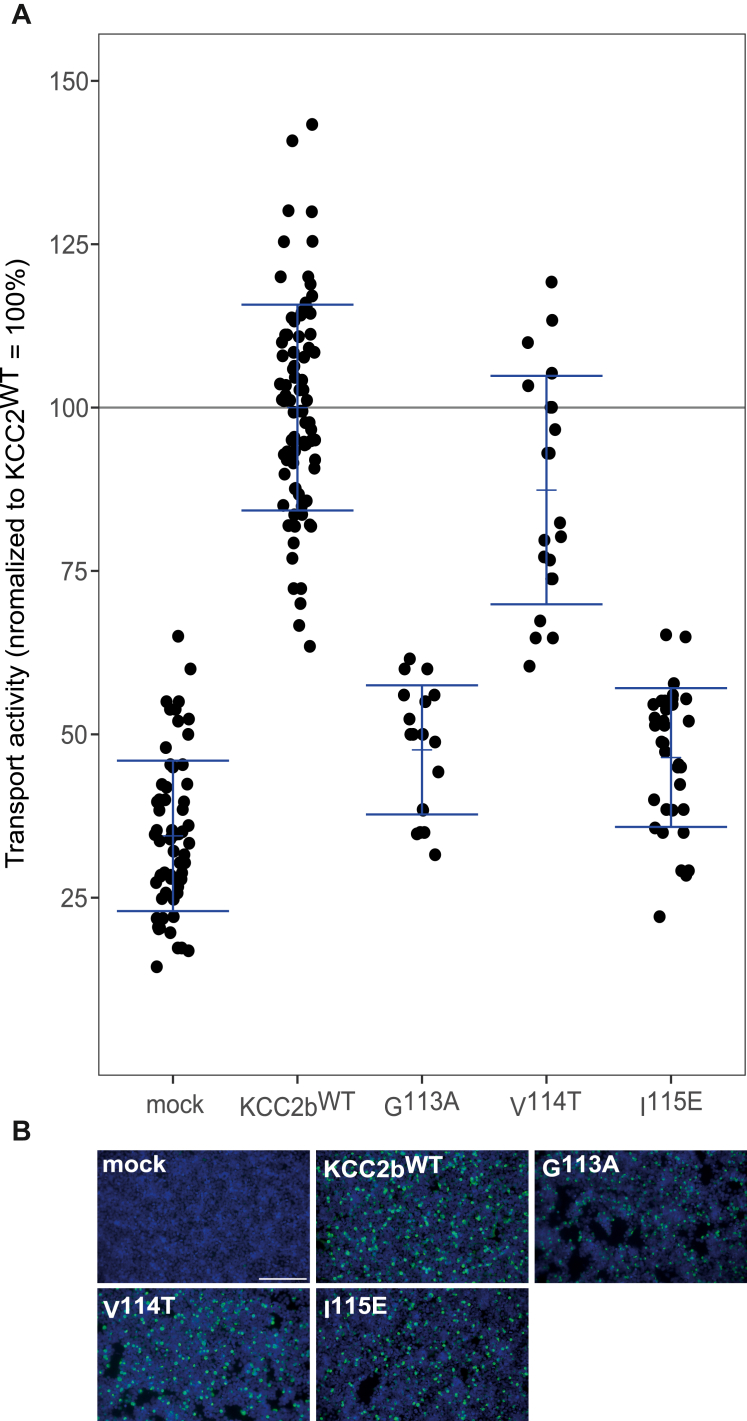
**Mutation of Cl**_**1**_^**−**^**-binding sites impairs KCC2b activity.** HEK-293 cells were transiently transfected with *rn*KCC2b^WT^ or *rn*KCC2 variants with mutations in the chloride-binding site 1. Cells were then seeded in parallel for Tl^+^ flux measurements and immunocytochemistry. *A*, Tl^+^ flux measurements were performed to determine the transport activity. The suggested Cl_1_ residue mutants in TM1 *rn*KCC2b^G113A^ (48% ± 10%, *p* = 2.11 × 10^−09^ compared with *rn*KCC2b^WT^), *rn*KCC2b^V114T^ (87% ± 17%, *p* = 0.0009 compared with *rn*KCC2b^WT^), and KCC2b^I115E^ (46% ± 11%, *p* = 5.65 × 10^−19^ compared with *rn*KCC2b) showed diminished KCC2 activity (Table 1). The graph represents the data of at least five independent measurements including three technical replicates per independent measurement, normalized to *rn*KCC2b^WT^. Statistical analysis can be seen in Table 1 (∗∗∗*p* < 0.001, two-sample *t* test with Benjamini–Hochberg adjustment for multiple comparisons). *B*, immunocytochemistry was used to monitor the transfection rate of the *rn*KCC2 variants (*green*) and cell staining by DAPI (*blue*). Representative immunocytochemical images were used for the biological replicates. The scale bar represents 200 μm. DAPI, 4′,6-diamidine-2-phenylindole; HEK-293, human embryonic kidney 293 cell line; KCC, K^+^–Cl^−^ cotransporter; *Rn*, Rattus norvegicus; TM, transmembrane helix.

**Figure 4:**
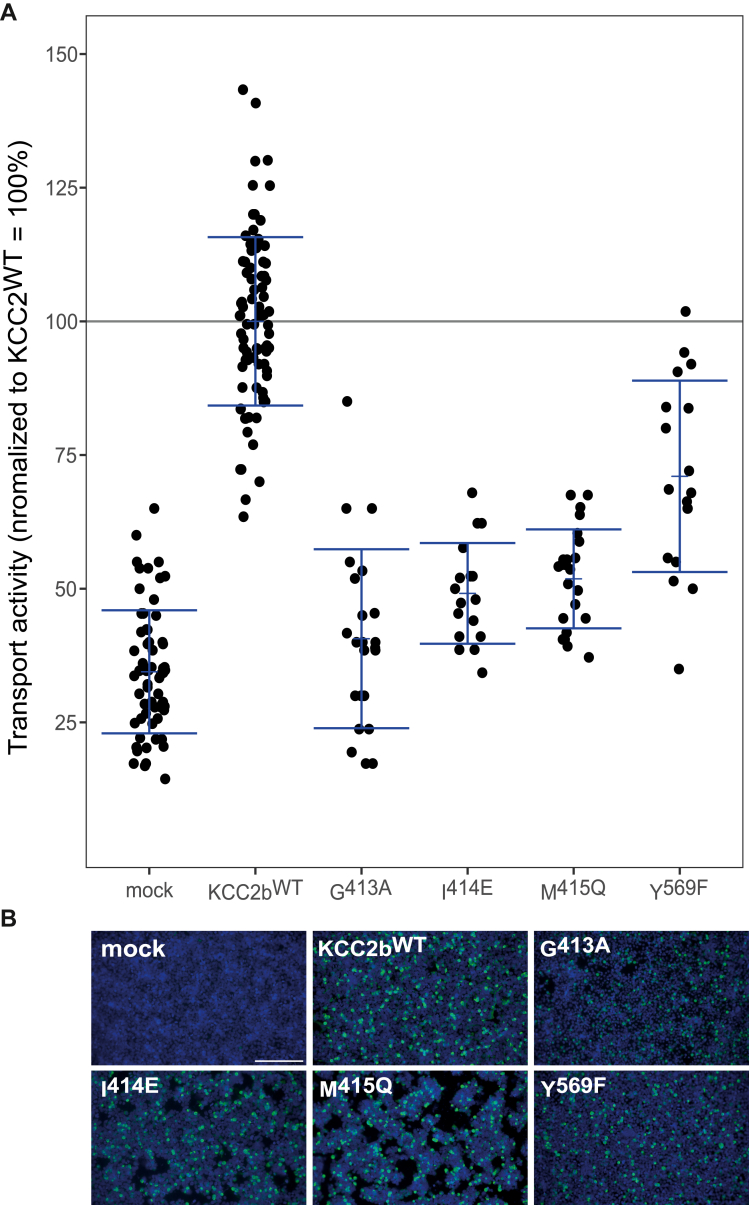
**Mutation of the Cl**^**−**^_**2**_**-binding site impairs KCC2b activity.** HEK-293 cells were transiently transfected with *rn*KCC2b^WT^ or *rn*KCC2b variants with mutations in the chloride-binding site 2. Cells were then seeded in parallel for Tl^+^ flux measurements and immunocytochemistry. *A*, Tl^+^ flux measurements were performed to determine the transport activity. The Tl^+^ flux determines abolished transport activity for *rn*KCC2b^G413A^ in TM6 (41% ± 17%, *p* = 0.13 compared with mock). *rn*KCC2b^I414E^ (49% ± 9%, *p* = 2.38 × 10^−9^ compared with *rn*KCC2b^WT^) and *rn*KCC2b^M415Q^ (52% ± 9%, *p* = 5.75 × 10^−14^ compared with *rn*KCC2b^WT^) in TM6 and *rn*KCC2b^Y569F^ (71% ± 18%, *p* = 8.04 × 10^−8^ compared with *rn*KCC2b^WT^) in TM10 diminished KCC2 transport activity (100% ± 16%) (Table 2). The graph represents the data of at least five independent measurements including three technical replicates per independent measurement, normalized to *rn*KCC2b^WT^. Statistical analysis can be seen in Table 2 (∗∗∗*p* < 0.001, two-sample *t* test with Benjamini–Hochberg adjustment for multiple comparisons). *B*, immunocytochemistry was used to monitor the transfection rate of the *rn*KCC2 variants (*green*) and cell staining by DAPI (*blue*). Representative immunocytochemical images were used for the biological replicates. Since the readings for mock and KCC2^wt^ of all Tl^+^-based activity measurements were combined, the representative figure for mock and KCC2^wt^ was used here as it is also available in Figure 3. The scale bar represents 200 μm. DAPI, 4′,6-diamidine-2-phenylindole; HEK-293, human embryonic kidney 293 cell line; KCC, K^+^–Cl^−^ cotransporter; *Rn*, Rattus norvegicus; TM, transmembrane helix.

**Supporting Figure S1:**
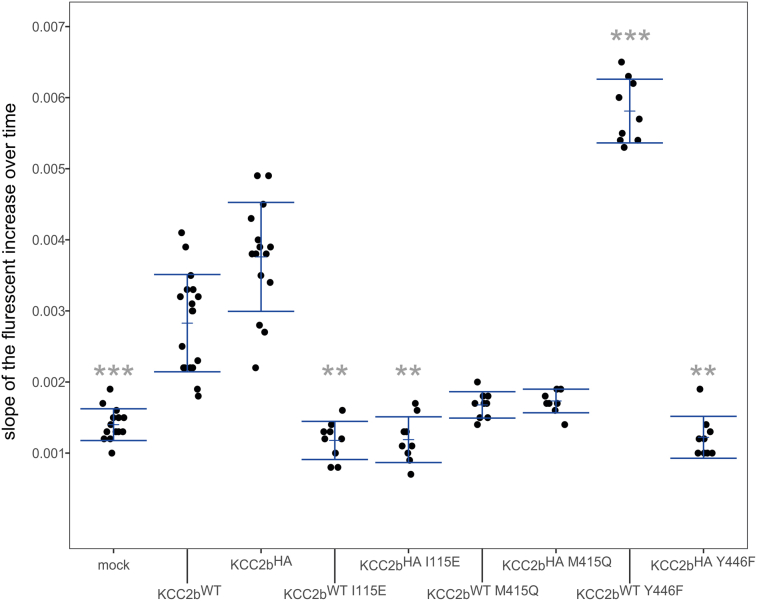


**Supporting Figure S2:**
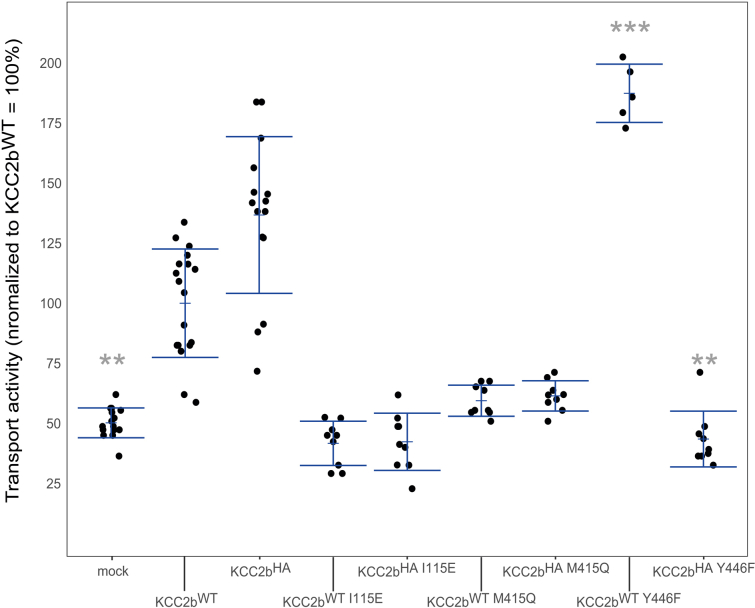


Recently published cryo-EM structures revealed that KCCs have one highly conserved K^+^ and two highly conserved Cl^−-^binding sites (Fig. 2*A*) (20, 23, 24, 27, 29, 35). We carefully investigated the structure of KCC2 (Protein Data Bank [PDB] code: 6M23) (24); however at the site, where the K^+^ ion has been modeled in, no electron density has been observed. To model K^+^ and Cl^−^ coordination sites in KCC2, we therefore used the structural homolog KCC4 as a model for graphical representation and structural analysis (PDB code: 7D99) (21). Cl_1_ is formed by main-chain interactions of glycine, valine, and isoleucine in TM1 and Tyr in TM3, whereas Cl_2_ is formed by main-chain interactions of glycine, isoleucine, and methionine in TM6 and side-chain interaction of tyrosine in TM10 (Fig. 2*A*) (20, 23, 24, 29). The center of Cl_1_ is 3.4 Å far away from the center of the K^+^ coordination, which opens the possibility of a direct coupling of these ions (Fig. 2*A*). Cl_2_ is 7.4 Å away from the coordination center of the K^+^ ion (Fig. 2*A*). This raises questions about the functional role of both Cl^−^ sites. To address this important question, residues of both Cl^−-^binding sites were mutated into residues of similar size but different chemical properties. This resulted in the following seven murine KCC2b mutations: *rn*KCC2b^G113A^, *rn*KCC2b^V114T^, *rn*KCC2b^I115E^, *rn*KCC2b^G413A^, *rn*KCC2b^I414E^, *rn*KCC2b^M415Q^, and *rn*KCC2b^Y569F^. All mutants showed transfection rates similar to *rn*KCC2b^WT^ in human embryonic kidney 293 (HEK-293) cells (Figs 3*B* and 4*B*). HEK-293 cells transiently expressing *rn*KCC2b^WT^ displayed a significantly higher KCC2 transport activity (100%) than mock-transfected control cells (34% ± 12%, Fig. 3*A*). The transport activity has been measured by Cl^−^-dependent uptake of Tl+ in HEK-293 cells as described previously (14, 59, 60). Afterward, the transport activity was normalized according to KCC2b^WT^ activity. Comparison of the absolute *versus* normalized values does not indicate negative effects of normalization (Figs. S1 and S2). The Cl_1_ residue mutants *rn*KCC2b^G113A^ (48% ± 10%, *p* = 2.11 × 10^−9^ compared with KCC2b^WT^), *rn*KCC2b^V114T^ (87% ± 17%, *p* = 0.0009), and *rn*KCC2b^I115E^ (46% ± 11%, *p* = 5.65 × 10^−19^) compared with *rn*KCC2b^WT^ showed diminished KCC2 activity (Fig. 3*A*. Table 1). These results confirm the importance of Gly^113^, Val^114^, and Ile^115^ in TM1 for Cl^−^ coordination at Cl_1_.

**Table 1:** Transport activity of Cl_1_-binding site mutations in KCC2b

Construct	Mean ± SD	Significance in comparison to *rn*KCC2b^WT^	Significance in comparison to mock
Mock	34% ± 12%	∗∗∗	—
*rn*KCC2b^WT^	100% ± 16%	—	∗∗∗
G113A	48% ± 10%	∗∗∗	∗∗∗
V114T	87% ± 17%	∗∗∗	∗∗∗
I115E	46% ± 11%	∗∗∗	∗∗∗

∗∗∗*p* = 0.001.

To analyze the importance of the suggested Cl_2_-binding sites for KCC2 function, we used the mutants *rn*KCC2b^G413A^, *rn*KCC2b^I414E^, and *rn*KCC2b^M415Q^ in TM6 and *rn*KCC2b^Y569F^ in TM10. The mutation KCC2b^G413A^ (41% ± 17%, *p* = 0.13 compared with mock) abolished KCC2 transport activity. The mutants *rn*KCC2b^I414E^ (49% ± 9%, *p* = 2.38 × 10^−9^), Met^415^ to glutamine (52% ± 9%, *p* = 5.75 × 10^−14^), and *rn*KCC2b^Y569F^ (71% ± 18%, *p* = 8 × 10^−8^) compared with *rn*KCC2b^WT^ showed reduced KCC2 transport activity, revealing a crucial role in KCC2 function (Fig. 4*A*. Table 2). Thus, Cl^−^ in Cl_2_ is coordinated by main-chain interactions of Gly^413^, Ile^414^, and Met^415^ in TM6 and side-chain interaction of Tyr^569^ in TM10. Most importantly, these data demonstrate that both Cl_1_ and Cl_2_ are required for proper KCC2 transport activity.

**Table 2:** Transport activity of Cl_2_-binding site mutations in KCC2b

Construct	Mean ± SD	Significance in comparison to *rn*KCC2b^WT^	Significance in comparison to mock
Mock	34% ± 12%	∗∗∗	—
*rn*KCC2b^WT^	100% ± 16%	—	∗∗∗
G413A	41% ± 17%	∗∗∗	n.s.
I414E	49% ± 9%	∗∗∗	∗∗∗
M415Q	52% ± 9%	∗∗∗	∗∗∗
Y569F	71% ± 18%	∗∗∗	∗∗∗

Abbreviation: n.s., nonsignificant.

∗∗∗*p* = 0.001

### Cl^−^-binding sites in KCC2b^HA^

Previously, we showed that an *mm*KCC2b variant with an insertion of a 3xHA tag in EL2 results in subtle conformational changes of ECD and switches the K^+^-binding site to an alternative K^+^-binding site predicted to consist of Pro^409^, Ser^410^ (in TM6), and Tyr^446^ (in TM10) (59). To investigate, whether this extension of EL2 also has an impact on the two Cl^−^-binding sites, we here generated the following seven mutants: *mm*KCC2b^HA-G113A^, *mm*KCC2b^HA-V114T^, *mm*KCC2b^HA-I115E^ for Cl_1_ and *mm*KCC2b^HA-G413A^, *mm*KCC2b^HA-I414E^, *mm*KCC2b^HA-M415Q^, and *mm*KCC2b^HA-Y569F^ for Cl_2_. The *mm*KCC2b^HA^ construct advantageously displays the same transport activity as *rn*KCC2b^WT^ and thus enables a systematic investigation of ion-binding sites *via* mutagenesis (59).

**Figure 5:**
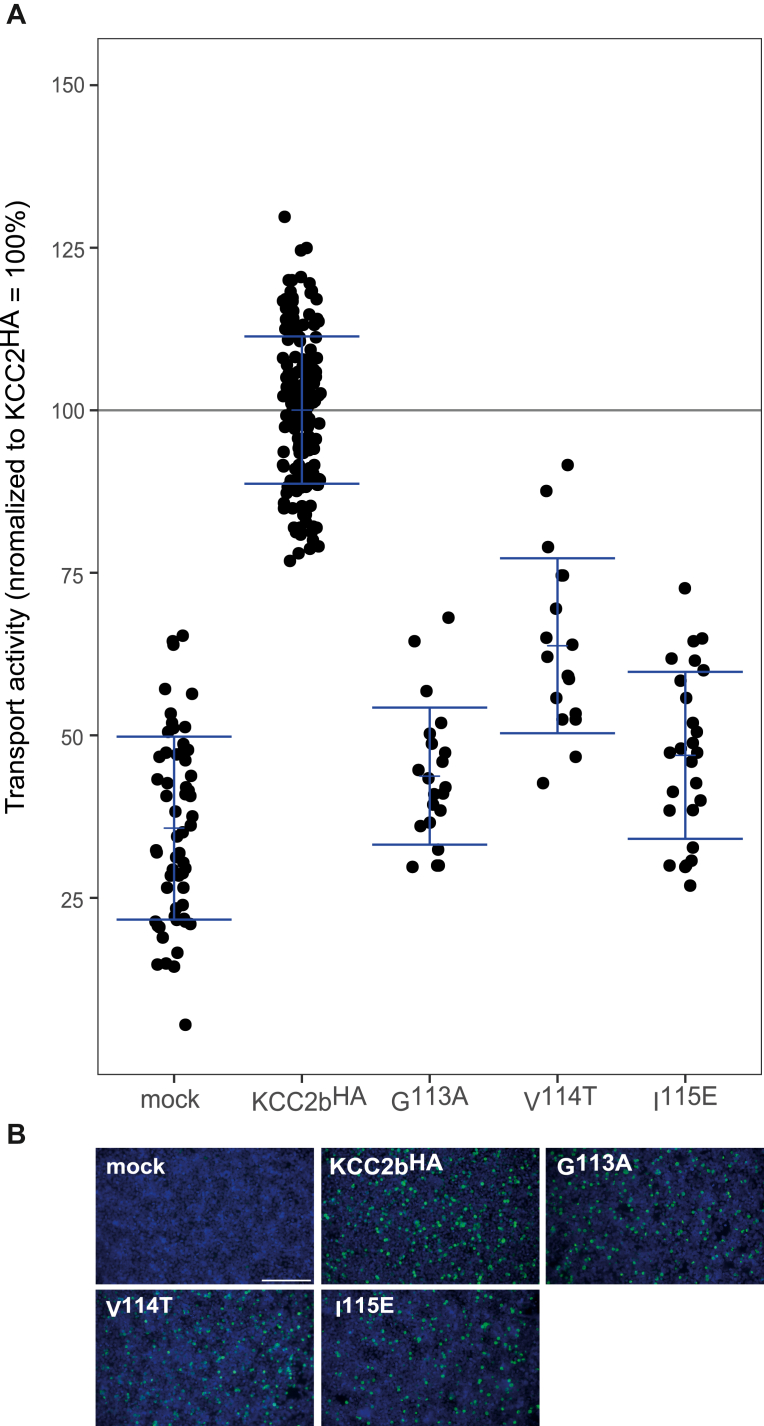
**Mutation of the Cl**^**−**^_**1**_**-binding site impairs KCC2b**^**HA**^**activity.** HEK-293 cells were transiently transfected with *mm*KCC2b^HA^ or *mm*KCC2b^HA^ variants with mutations in the chloride-binding site 1. Cells were then seeded in parallel for Tl^+^ flux measurements and immunocytochemistry. *A*, Tl^+^ flux measurements were performed to determine the transport activity. The Tl^+^ flux determines for *mm*KCC2b^HA-G113A^ (44% ± %, *p* = 0.026 compared with mock) and *mm*KCC2^HA-I115E^ (47% ± 13%, *p* = 0.02 compared with mock) in TM1 show abolished transport activity. *mm*KCC2b^HA-V114T^ (64% ± 13%, *p* = 1.91 × 10^−18^ compared with *mm*KCC2b^HA^) showed diminished transport activity compared with KCC2b^HA^ (100% ± 11%). The graph represents the data of at least five independent measurements including three technical replicates per independent measurement, normalized to *mm*KCC2b^HA^. Statistical analysis can be seen in Table 3 (∗∗∗*p* < 0.001, two-sample *t* test with Benjamini–Hochberg adjustment for multiple comparisons). *B*, immunocytochemistry was used to monitor the transfection rate of the *mm*KCC2^HA^ variants (*green*) and cell staining by DAPI (*blue*). Representative immunocytochemical images were used for the biological replicates. Since the readings for mock of all Tl^+^-based activity measurements were combined, the representative figure for mock was used here as it is also available in Figure 3. The scale bar represents 200 μm. DAPI, 4′,6-diamidine-2-phenylindole; HEK-293, human embryonic kidney 293 cell line; KCC, K^+^–Cl^−^ cotransporter; *Mm*, *Mus musculus*; TM, transmembrane helix.

All mutants showed similar transfection rates in HEK-293 compared with *mm*KCC2b^HA^ (Fig. 5*B*). HEK-293 cells transiently expressing *mm*KCC2b^HA^ displayed a significantly higher KCC2 transport activity (100% ± 11%) than mock-transfected control cells (36% ± 14%, Figs. 4*A*, and 5*A*). The Cl_1_ mutation *mm*KCC2b^HA-G113A^ (44% ± 11%, *p* = 0.026 compared with mock) and *mm*KCC2b^HA-I115E^ (47% ± 13%, *p* = 0.002 compared with mock) abolished and *mm*KCC2b^HA-V114T^ (64% ± 13%, *p* = 1.9 × 10^−18^ compared with *mm*KCC2b^HA^) diminished KCC2 transport activity (Fig. 5*A*, Table 3). These results are in line with the mutational analyses in KCC2^WT^ and corroborate the importance of these Cl^−^ binding-sites in Cl_1_ for KCC2 function. In addition, these data identify a fundamental difference with regard to the K^+^ coordination site, which is affected (59).

**Table 3:** Transport activity of Cl_1_-binding site mutations in KCC2b^HA^

Construct	Mean ± SD	Significance in comparison to *mm*KCC2b^HA^	Significance in comparison to mock
Mock	36% ± 14%	∗∗∗	—
*mm*KCC2b^HA^	100% ± 11%	—	∗∗∗
G113A	44% ± 11%	∗∗∗	n.s.
V114T	64% ± 13%	∗∗∗	∗∗∗
I115E	47% ± 13%	∗∗∗	n.s.

Abbreviation: n.s., nonsignificant.

∗∗∗*p* = 0.001.

**Figure 6:**
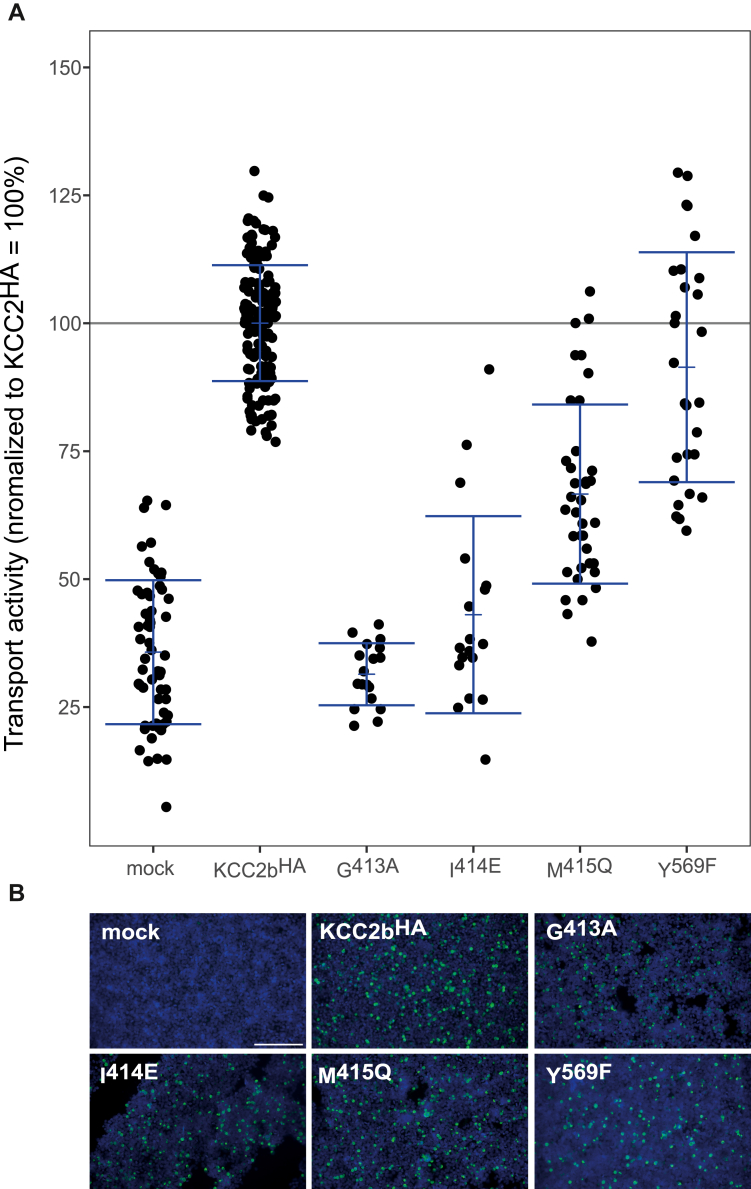
**Mutation of the Cl**^**−**^_**2**_**-binding sites impairs KCC2b**^**HA**^**transport activity.** HEK-293 cells were transiently transfected with *mm*KCC2b^HA^ or *mm*KCC2b^HA^ variants with mutations in the chloride-binding site 2. Cells were then seeded in parallel for Tl^+^ flux measurements and immunocytochemistry. *A*, Tl^+^ flux measurements were performed to determine the transport activity. The Tl^+^ flux measurements showed that *mm*KCC2b^HA-G413A^ (31% ± 6%, *p* = 0.09 compared with mock) and *mm*KCC2b^HA-I414E^ (43% ± 19%, *p* = 0.09 compared with mock) abolished KCC2 transport activity. Substitution of *mm*KCC2b^HA-M415Q^ diminished KCC2 transport activity (67% ± 18%, *p* = 8.35 × 10^−8^ compared with *mm*KCC2b^HA^). Contrary, *mm*KCC2b^HA-Y569F^ in TM10 (91% ± 22%, *p* = 0.08 compared with *mm*KCC2b^HA^) showed no significant impairment of transport activity compared with KCC2b^HA^ (100% ± 11%). The graph represents the data of at least five independent measurements including three technical replicates per independent measurement, normalized to *mm*KCC2b^HA^. Statistical analysis can be seen in Table 4 (∗∗∗*p* < 0.001, two-sample *t* test with Benjamini–Hochberg adjustment for multiple comparisons). Representative immunocytochemical images were used for the biological replicates. Since the readings for mock of all Tl^+^-based activity measurements were combined, the representative figure for mock and KCC2^HA^ was used here as it is also available in Figure 3 (for mock) and Figure 5 (for KCC2^HA^). *B*, immunocytochemistry was used to monitor the transfection rate of the *mm*KCC2^HA^ variants (*green*) and cell staining by DAPI (*blue*). The scale bar represents 200 μm. DAPI, 4′,6-diamidine-2-phenylindole; HEK-293, human embryonic kidney 293 cell line; KCC, K^+^–Cl^−^ cotransporter; *Mm*, *Mus musculus*; TM, transmembrane helix.

For Cl_2_, the mutants *mm*KCC2b^HA-G413A^ (31% ± 6%, *p* = 0.09 compared with mock) and *mm*KCC2b^HA-I414E^ (43% ± 19%, *p* = 0.09 compared with mock) abolished KCC2 transport activity (Fig. 6*A*, Table 4), and substitution of Met^415^ to glutamine diminished KCC2b^HA^ activity (67% ± 18%, *p* = 8.85 × 10^−8^ compared with KCC2b^HA^). These results are in line with the analyzed Cl^−^-binding sites in Cl_2_ for KCC2^WT^ (see aforementioned). Contrary to *rn*KCC2b^Y569F^, the mutant *mm*KCC2b^HA-Y569F^ (91% ± 22%, *p* = 0.08 compared with KCC2b^HA^) did not alter KCC2 transport activity. Thus, the extension of the EL2 with the 3xHA tag affects side-chain interaction of Tyr but not main-chain interactions of Gly, Ile, and Met with Cl^−^ in Cl_2_.

**Table 4:** Transport activity of Cl_2_-binding site mutations in KCC2b^HA^

Construct	Mean ± SD	Significance in comparison to *mm*KCC2b^HA^	Significance in comparison to mock
Mock	36% ± 14%	∗∗∗	—
*mm*KCC2b^HA^	100% ± 11%	—	∗∗∗
G413A	31% ± 6%	∗∗∗	n.s.
I414E	43% ± 19%	∗∗∗	n.s.
M415Q	67% ± 18%	∗∗∗	∗∗∗
Y569F	91% ± 22%	n.s.	∗∗∗

Abbreviation: n.s., nonsignificant.

∗∗∗*p* = 0.001.

### Alternative K^+^-binding sites in KCC2b^HA^

**Figure 7:**
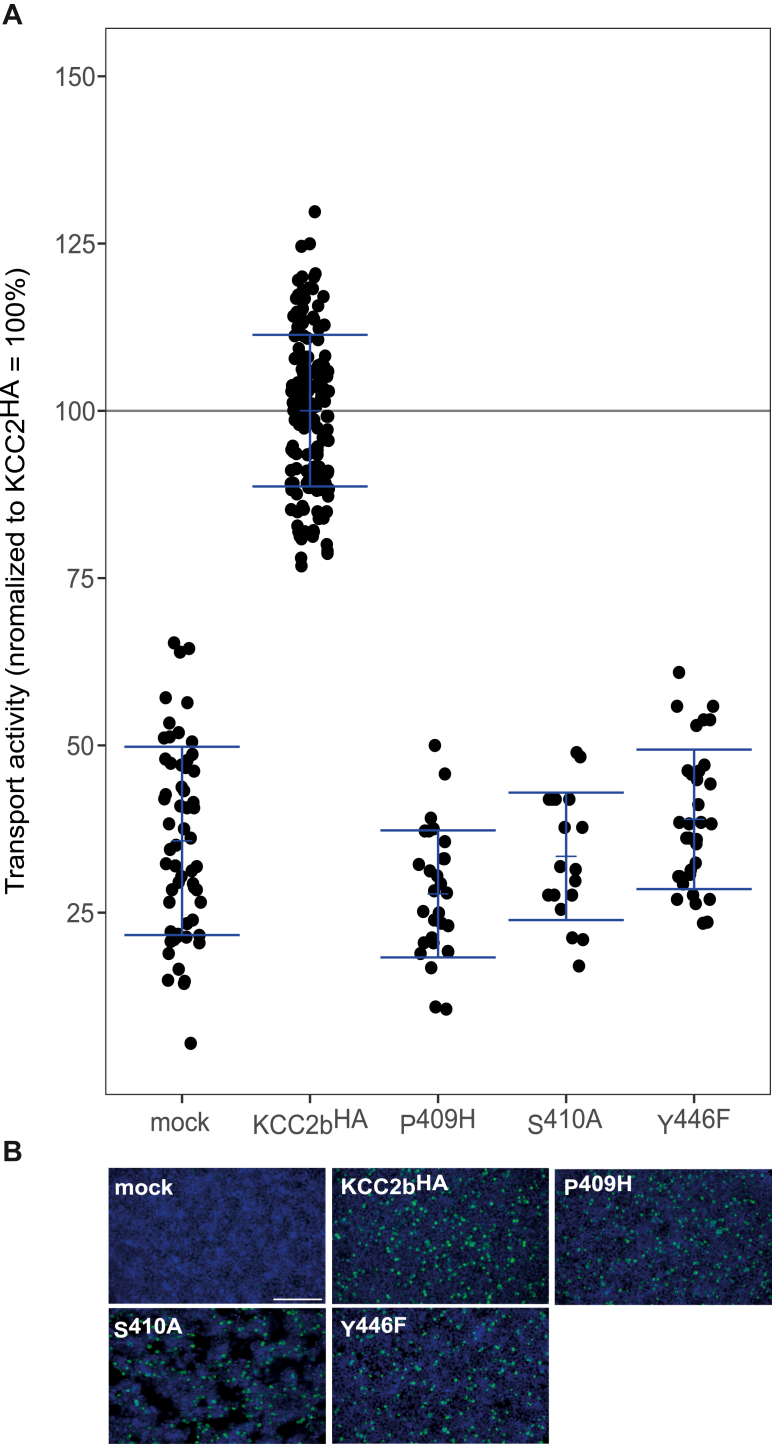
**Mutations of the alternative K**^**+**^**-binding site in KCC2b**^**HA**^**abolishes KCC2 activity.** HEK-293 cells were transiently transfected with *mm*KCC2b^HA^ or *mm*KCC2b^HA^ variants with mutations in the alternative K^+^. Cells were then seeded in parallel for Tl^+^ flux measurements and immunocytochemistry. *A*, Tl^+^ flux measurements were performed to determine the transport activity. The constructs *mm*KCC2b^HA-P409H^ (30% ± 13%, *p* = 0.22 compared with mock), *mm*KCC2b^HA-S410A^ (33% ± 10%, *p* = 0.44 compared with mock), and *mm*KCC2b^HA-Y446F^ (39% ± 10%, *p* = 0.44 compared with mock) abolished transport activity (100% ± 11%). The graph represents the data of at least five independent measurements including three technical replicates per independent measurement, normalized to *mm*KCC2b^HA^. Statistical analysis can be seen in Table 5 (∗∗∗*p* < 0.001, two-sample *t* test with Benjamini–Hochberg adjustment for multiple comparisons). *B*, immunocytochemistry was used to monitor the transfection rate of the *mm*KCC2^HA^ variants (*green*) and cell staining by DAPI (*blue*). Representative immunocytochemical images were used for the biological replicates. Since the readings for mock of all Tl^+^-based activity measurements were combined, the representative figure for mock and KCC2^HA^ was used here as it is also available in Figure 3 (for mock) and Figure 5 (for KCC2^HA^). The scale bar represents 200 μm. DAPI, 4′,6-diamidine-2-phenylindole; HEK-293, human embryonic kidney 293 cell line; KCC, K^+^–Cl^−^ cotransporter; *Mm*, *Mus musculus*; TM, transmembrane helix.

In the *mm*KCC2b^HA^ construct, we previously hypothesized a switch from Asn^110^, Ile^111^ (in TM1), Tyr^195^ (in TM3), Pro^409^, and Thr^412^ (in TM6) K^+^-binding sites to the predicted alternative K^+^-binding site consisting of Pro^409^, Ser^410^ (in TM6), and Tyr^446^ (in TM10) (Fig. 2*B*) (59). To investigate the role of these alternative residues, we generated the following constructs *via* site-directed mutagenesis: *mm*KCC2b^HA-P409H^, *mm*KCC2b^HA-S410A^, and *mm*KCC2b^HA-Y446F^. All mutants showed transfection rates in HEK-293 cells similar to *mm*KCC2b^HA^ (Fig. 7*B*). HEK-293 cells transiently expressing KCC2b^HA^ displayed a significantly higher KCC2 transport activity (100%) than mock-transfected control cells (36% ± 14%, *p* = 3.43 × 10^−46^ Fig. 7*A*). The constructs *mm*KCC2b^HA-P409H^ (30% ± 13%, *p* = 0.23 compared with mock), *mm*KCC2b^HA-S410A^ (33% ± 10%, *p* = 0.44 compared with mock), and *mm*KCC2b^HA-Y446F^ (39% ± 10%, *p* = 0.44 compared with mock) abolished KCC2 transport activity (Fig. 7, Table 5). These data confirm the hypothesis that the extension of EL2 with a 3xHA tag results in subtle conformational changes of the ECD, which switches the K^+^-binding site to the alternative K^+^-binding site Pro^409^, Ser^410^, and Tyr^446^.

**Table 5:** Transport activity of alternative K^+^-binding site mutations in KCC2b^HA^

Construct	Mean ± SD	Significance in comparison to *mm*KCC2b^HA^	Significance in comparison to mock
Mock	36% ± 14%	∗∗∗	—
*mm*KCC2b^HA^	100% ± 11%	—	∗∗∗
P409H	30% ± 13%	∗∗∗	n.s.
S410A	33% ± 10%	∗∗∗	n.s.
Y446F	39% ± 10%	∗∗∗	n.s.

Abbreviation: n.s., nonsignificant.

∗∗∗*p* = 0.001.

**Figure 8:**
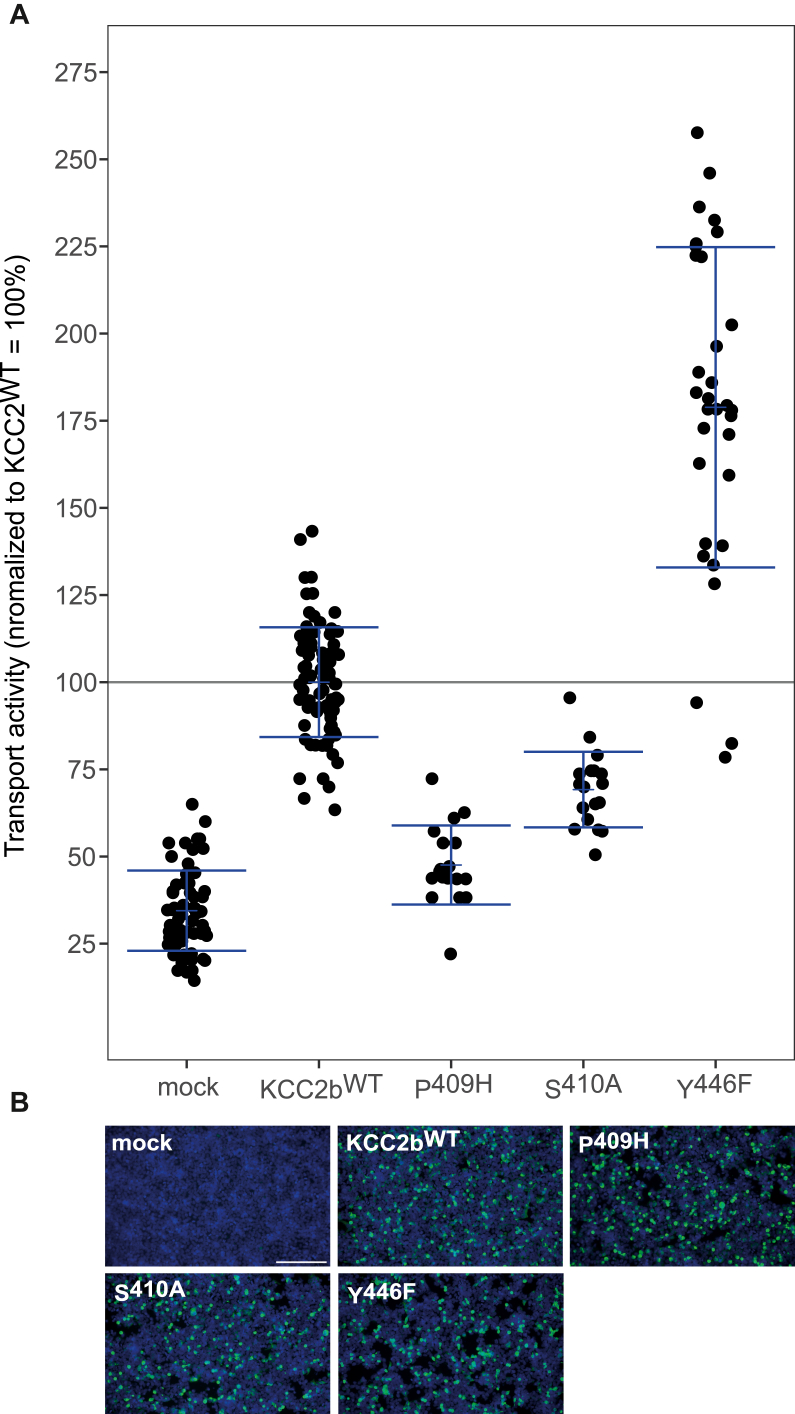
**Mutations of the alternative K**^**+**^**binding in KCC2b differentially affect KCC2 function.** HEK-293 cells were transiently transfected with *rn*KCC2b^WT^ or *rn*KCC2b variants with mutations in the alternative K^+^. Cells were then seeded in parallel for Tl^+^ flux measurements and immunocytochemistry. *A*, Tl^+^ flux measurements were performed to determine the transport activity. The constructs *rn*KCC2b^P409H^ (48% ± 11%, *p* = 2.39 × 10^−14^ compared with *rn*KCC2b^WT^) and *rn*KCC2b^S410A^ (69% ± 11%, *p* = 2.15 × 10^−6^ compared with *rn*KCC2b^WT^) diminished KCC2 transport activity, whereas *rn*KCC2b^Y446F^ (188% ± 59%, *p* = 5.93 × 10^−6^ compared with *rn*KCC2b^WT^) enhanced KCC2 activity. The graph represents the data of at least five independent measurements including three technical replicates per independent measurement, normalized to *rn*KCC2b^WT^. Statistical analysis can be seen in Table 6 (∗∗∗*p* < 0.001, two-sample *t* test with Benjamini–Hochberg adjustment for multiple comparisons). *B*, immunocytochemistry was used to monitor the transfection rate of the *rn*KCC2 variants (*green*) and cell staining by DAPI (*blue*). Representative immunocytochemical images were used for the biological replicates. Since the readings for mock and KCC2^wt^ of all Tl^+^-based activity measurements were combined, the representative figure for mock and KCC2^wt^ was used here as it is also available in Figure 3. The scale bar represents 200 μm. DAPI, 4′,6-diamidine-2-phenylindole; HEK-293, human embryonic kidney 293 cell line; KCC, K^+^–Cl^−^ cotransporter; *Rn*, Rattus norvegicus; TM, transmembrane helix.

To test how equivalent mutations in *rn*KCC2b affect its transport activity, we generated the mutations: *rn*KCC2b^P409H^, *rn*KCC2b^S410A^, and *rn*KCC2b^Y446F^ in *rn*KCC2b^WT^. All mutants showed similar transfection rates in HEK-293 compared with *rn*KCC2b^WT^ (Fig. 8*B*). HEK-293 cells transiently expressing KCC2b displayed a significantly higher KCC2 transport activity (100% ± 16%, *p* = 2.23 × 10^−30^) than mock-transfected control cells (34% ± 12%, *p* = 2.23 × 10^−30^; Fig. 8*A*, Table 6). The constructs *rn*KCC2b^P409H^ (48% ± 11%, *p* = 2.39 × 10^−14^ compared with KCC2b^WT^) and *rn*KCC2b^S410A^ (69% ± 11%, *p* = 2.15 × 10^−6^ compared with KCC2b^WT^) showed diminished transport activities, whereas *rn*KCC2b^Y446F^ (188% ± 59%, *p* = 5.93 × 10^−6^ compared with *rn*KCC2b^WT^) enhanced KCC2 activity (Fig. 8*A*, Table 6). Thus, Pro^409^ likely is important for coordinating K^+^ at the “normal” K^+^-binding site as well as at the alternative K^+^ site. We previously showed that mutation of Pro^432^ to histidine in *mm*KCC2a diminished KCC2 transport activity (59). This is in line with our results here and underpin its general role in K^+^ coordination. The reduced activity of *rn*KCC2b^S410A^ may be explained by a weak coordinating role of Ser^410^ at Cl_1_. Mutations of Ser^430^ in KCC1 and Ser^495^ in KCC3 to alanine have already been shown to reduce KCC activity (29). The only residue that showed a strikingly completely different property is Tyr^446^. In *mm*KCC2b^HA^, mutation of Tyr^446^ to phenylalanine abolished KCC2 activity, whereas Tyr^446Phe^ in *rn*KCC2b enhanced KCC2 activity. This confirms that Tyr^446^ plays a crucial role in coordinating K^+^ at the alternative K^+^ site. To fully ensure that the insertion of the HA tag leads to a shift of the K^+^ coordination site here, a cryo-EM structure of KCC2^HA^ is required.

**Table 6:** Transport activity of alternative K^+^-binding site mutations in KCC2b

Construct	Mean ± SD	Significance in comparison to *rnKCC2b*^*WT*^	Significance in comparison to mock
Mock	34% ± 12%	∗∗∗	—
*rn*KCC2b^WT^	100% ± 16%	—	∗∗∗
P409H	48% ± 11%	∗∗∗	∗∗∗
S410A	69% ± 11%	∗∗∗	∗∗∗
Y446F	188% ± 59%	∗∗∗	∗∗∗

Abbreviation: n.s., nonsignificant.

∗∗∗*p* = 0.001.

## Discussion

Recently published structural analyses of CCCs by cryo-EM revealed evolutionary highly conserved K^+^ and Cl^−^ coordinating sites (15, 20, 21, 23, 27, 28, 29, 30, 31, 34, 35, 36). Although KCCs transport K^+^ and Cl^−^ in a stoichiometric electroneutral ratio of 1:1, one K^+^ and two Cl^−^ coordinating sites were revealed (20, 23, 29, 31, 33). The cryo-EM structure of KCC1 was the first structural analysis to show that two Cl^−^ coordination sites are present, which was previously not been unambiguously shown (29). This raises the question of whether both Cl^−^ coordinating sites are essential for KCC2 function.

Mutation of Tyr in TM10 that coordinates binding of Cl^−^ in Cl_2_ reduces or abolishes the activity of KCC1, KCC3, KCC4, NKCC1, and NCC indicating that the Cl_2_ site is important for their function (27, 28, 29, 35). Here, we unambiguously demonstrated that mutation of residues coordinating Cl^−^ in Cl_1_ (Gly^113^, Val^114^, and Ile^115^ in TM1) or Cl_2_ (Gly^413^, Ile^414^, Met^415^ in TM6 and Tyr^569^ in TM10) significantly reduced or abolished KCC2 activity. In addition, Tyr^195^ in TM3 has a bifunctional role in stabilizing K^+^ and Cl^−^ in Cl_1_ (20). Mutation of Tyr^195^ reduced KCC2 activity indicating a crucial role in coordinating both ions (59). Analogous experiments in KCC1 (Tyr^216^), KCC3 (Tyr^283^), or KCC4 (Tyr^216^) support this hypothesis (27, 28, 29). Thus, both suggested that Cl^−-^binding sites are important for KCC2 function. This impairment of the transport activity likely results from intrinsic conformational changes. However, we cannot exclude a contribution of decreased cell surface abundance of KCC2 to impaired transport activity but consider this unlikely.

The requirement of both Cl^−-^binding sites and their evolutionary conservation in KCCs is enigmatic since they transport K^+^ and Cl^−^ in a 1:1 ratio. One explanation is that one binding site is important for K^+^-coupled Cl^−^ transport and the other Cl^−^ site “allosterically” affects the binding of the other ions (23, 29, 58). Since the K^+^ and the Cl_1_-binding sites are within 3.4 Å and thus in close proximity, it was hypothesized that ions coordinate with each other to transport both ions in a stoichiometric ratio (Fig. 2*A*) (29, 58). In contrast, the center of Cl_2_ is 7.4 Å away from the coordination center of the K^+^ ion. Therefore, a direct coupling of K^+^ and Cl^−^ in Cl_2_ is not given here (Fig. 2*A*). In this scenario, the Cl_2_ site has an “allosteric” effect in stabilizing the coordination of the K^+^-binding site (29, 58). In addition, the binding of Cl^−^ at Cl_2_ may strengthen the interactions between TM6a+b and TM10 to couple conformational changes during ion translocation (29, 58). This scenario is supported by MD simulations in KCC1 (29). Another scenario suggested that Cl_2_ presents the transporting site (28). Considering that there is only one universal Cl^−-^binding site in CCCs, it was assumed that this is not the Cl_1_ site, since NCC does not transport K^+^, and therefore, coupling the transport of K^+^ and Cl^−^ at Cl_1_ is not necessary (28). However, the recent cryo-EM structure of NCC showed that NCC has two Na^+^ and two Cl^−^ coordinating sites, which ensure a ratio of 1:1 (35). It is therefore possible that KCCs also have a second previously undetected K^+^ site, which then ensures a 1:1 ratio.

KCCs mainly serve as K^+^ and Cl^−^ extruders. Yet, under nonphysiological conditions (high extracellular [K^+^]), they can change transport direction to load cells with K^+^ and Cl^−^ (60). It is hence also possible that both Cl^−^ sites can act as a transporting or “allosteric” site depending on the transport direction. This aspect is supported by the fact that an asymmetry of ion binding in outward- or inward-mediated ion transport was observed (58, 61). In the physiological outward-mediated transport direction, intracellular ions bind randomly (58, 61), likely because of the close proximity of K^+^ and Cl^−^ at the Cl_1_ site. Binding of Cl^−^ to Cl_2_ then induces a conformational change required for ion translocation. In contrast, in the nonphysiological inward-mediated transport direction, extracellular ions bind in a determined order (Cl^−^ → K^+^) (58, 61). Here, Cl^−^ might bind first at Cl_2_ before binding of K^+^ and Cl^−^ at the Cl_1_ site. Binding of Cl^−^ at site 1 (allosteric site) may then induce a conformation that facilitates ion translocation. In our present analyses, we investigated the inward-mediated transport of Tl^+^ (congener of K^+^) and Cl^−^.

To take a closer look to the function of both Cl^−^ sites, we used a modified KCC2 construct, in which the EL2 is extended by a 37-amino acid-long HA tag. This modification, which importantly does not compromise transport activity, results in subtle conformational changes of ECD, which is transduced to TM1 and TM6 (59). These changes most probably alter K^+^-binding sites in KCC2^HA^ (59). Because of this conformational change, the K^+^-binding site switches to an alternative K^+^-binding site consisting of Pro^409^ and Ser^410^ in TM6 and Tyr^446^ in TM7 instead of Asn^110^, Tyr^195^ (TM1), and Thr^412^ (TM3) (Fig. 2, *A* and *B*) (59). K^+^ therefore moves toward Tyr^446^, which is then 3.5 Å far away from Tyr^446^ and thus is able to stabilize the K^+^ together with the other coordination sites (Fig. 2*B*). Mutation of these alternative residues in the KCC2^HA^ variant abolished KCC2 activity, which corroborates the presence of an alternative K^+^-binding site in KCC2^HA^.

In order to find out whether the extension of the EL2 with the 3xHA tag also affects the coordination of the two Cl^−^ coordination sites, we carried out the analogous experiments for both Cl^−^ coordinating sites in the KCC2^HA^ construct.

Residues that coordinate Cl^−^ in the Cl_1_ site are in close proximity to the K^+^-binding site. Thus, both ions are coupled with each other with a 3.4 Å distance between them (Fig. 2*A*). The extension of EL2 with the 3xHA tag shifts the K^+^ coordination site from Tyr^195^ in TM3 in KCC2^WT^ to Tyr^446^ in TM7 in KCC2^HA^ (59). However, residues coordinating Cl^−^ at Cl_1_ in KCC2^HA^ remain at the suggested Cl^−^ coordination positions. Although the K^+^ coordination sites shifted and the Cl_1_ coordinating sites remain at the same position, both ions are in close proximity and the distance between them is 2.9 Å (Fig. 2*B*). Since K^+^ and the alternative K^+^ site are both in close proximity to Cl^−^ in Cl_1_, coupling of both ions is present in both KCC2^WT^ and KCC2^HA^. Interestingly, in the K^+^-independent NCC, mutation of Tyr^386^ to alanine (analogous to Tyr^446^ in KCC2 [TM7]) abolishes its function (35). In NCC, Tyr^386^ coordinates Cl^−^ at the Cl_1_ site through a water molecule (35). Tyr^386^ also interacts with Ile^150^ and Ser^350^ from the broken helices of TM1 and TM6 to mediate conformational coupling during ion coordination (35). This underlines a central role of Tyr^446^ in coordination of K^+^ and Cl^−^ in KCC2^HA^ and likely in the coupling mechanism. Thus, subtle conformational changes in the ECD directly affect K^+^ coordination and slightly Cl^−^ coordination in Cl_1_, wherein a coupling of both ions is still given. Undoubtedly, it is of great interest to analyze the structure of KCC2^HA^ by MD simulations, but unfortunately, no experimental data for the structure of KCC2^HA^ are presently available. Moreover, the MD simulations by Zhang *et al.* (24) on the available cryo-EM structure of KCC2^WT^ were performed over 1 μs, which would be a publication on its own legitimacy and therefore beyond the scope of this study.

Similar to KCC2b, mutations of Gly^413^, Ile^414^, and Met^415^ in TM6 of KCC2^HA^ significantly reduced or abolished transport, indicating that main-chain interactions of these residues are important for Cl^−^ binding. In contrast, a difference exists for Tyr^569^ in TM10 between both constructs, as its mutation reduced transport activity in KCC2b but not in KCC2^HA^. The most likely scenario is that the HA tag–induced conformational changes that are transduced to TM1 and TM6 result in changes in the spatial localization of TM6 relative to TM10. Whereas residues in TM6 still coordinate Cl^−^, the residue coordinating Cl^−^ in TM10 switches to an alternative position. This results overall in a slightly altered Cl_2_ coordination site.

In summary, our data showed that both Cl^−^ coordination sites are important for KCC2 transport activity. Furthermore, the conformational changes in the ECD by interactions with other extracellular loops affect the coordination of both K^+^ and Cl^−^. Note, however, that the effect on the Cl^−^-binding sites is subtler.

## Experimental procedures

### Graphical representation of K^+^ and Cl^−^ coordination sites

Structural figures have been prepared using CCP4mg (62). Manual alternation of the K^+^-binding site in the PDB file (7D99) has been performed with Coot (63) after careful visual inspection of the electron density map. The alternative K^+^-binding site would be suitable to accommodate this ion without any obvious clashes and a minimum distance of 2.5 Å to the nearest atom in the surrounding.

### Construction of the expression clones

Site-directed mutagenesis of rat KCC2b (NM_134363.1) and mouse KCC2b (NM_020333.2) with an HA tag in the extracellular loop 2 3xHA tag (IFYPYDVPDYAGYPYDVPDYAGSYPYDVPDYAAHAAA) was performed according to the QuikChange mutagenesis system (Stratagene) (16, 64, 65). Forward oligonucleotides for the generation of the mutations are given in Tables 7 and 8. All generated clones were verified by sequencing (LGC Genomics).

**Table 7:** Forward primers used for site-directed mutagenesis in *rn*KCC2b and *mm*KCC2b of the chloride-binding sites

Constructs	Sequence 5′ to 3′
*rn*KCC2b G113A	CAGAACATCTTTGCTGTTATCCTCTTT
*rn*KCC2b V114T	AACATCTTTGGTACTATCCTCTTTCTG
*rn*KCC2b I115E	ATCTTTGGTGTTGAGCTCTTTCTGCGG
*mm*KCC2b G113A	CAGAACATCTTTGCTGTCATCCTCTTC
*mm*KCC2b V114T	AACATCTTTGGTACCATCCTCTTCCTG
*mm*KCC2b I115E	ATCTTTGGTGTCGAGCTCTTCCTGCGG
*rn*KCC2b G413A	TCAGTCACAGCGATCATGGCT
*rn*KCC2b I414E	AGTCACAGGGGAAATGGCTGG
*rn*KCC2b M415Q	TCAGTCACAGGGATCCAAGCTGGC
*rn*KCC2b Y569F	TTCCTGATGTGTTTCATGTTTGTGAAC
*mm*KCC2b M415Q	ACAGGGATCCAAGCTGGCTC
*mm*KCC2b Y569F	TTCCTAATCTGTTTCATGTTTGTGAAC

**Table 8:** Forward primers used for site-directed mutagenesis in *rn*KCC2b and *mm*KCC2b of the alternative K^+^-binding site

Constructs	Sequence 5′ to 3′
*rn*KCC2b P409H	GCATCTATTTCCACTCAGTCACAG
*rn*KCC2b S410A	ATCTATTTCCCCGCAGTCACAGGGAT
*rn*KCC2b/*mm*KCC2b Y446F	TCTGCTCTGTTCATCAGCTCTGTT
*mm*KCC2b P409H	GTATCTACTTCCACTCAGTCACAG
*mm*KCC2b S410A	TATCTACTTCCCCGCAGTCACAGGGAT

### Cell culturing

For immunocytochemistry and measurement of K^+^–Cl^−^ cotransporter activity, HEK-293 cells were transiently transfected with the respective constructs, using Turbofect (Fermentas). Cells were seeded in a 6-well plate 24 h prior to transfection. The Dulbecco's modified Eagle's medium was replaced 4 h before transfection. About 150 μl Opti-MEM (Invitrogen), 6 μl Turbofect, and the corresponding DNA amount were thoroughly mixed and incubated for 20 min at room temperature (RT). The mixture was applied to the cells that were then shaken at 300 rpm for 10 min at RT.

For K^+^–Cl^−^ cotransporter activity measurements, transfected HEK-293 cells were plated at a concentration of 1 × 10^5^ cells/well in a 0.1 mg/ml poly-l-lysine-coated black 96-well culture dish (Greiner Bio-One 24 h after transfection. Each transfected construct was plated out three times and represents three technical replicas. For the generation of independent biological replicates, the constructs were transfected separately. The remaining cells were plated on 0.1 mg/ml poly-l-lysine-coated glass coverslips. After ∼18 h, coverslips were proceeded for immunocytochemical analysis to determine transfection rates, which were routinely between 20 and 30%.

### Immunocytochemistry

For immunocytochemistry, all steps were performed at RT. HEK-293 cells grown on poly-l-lysine-coated coverslips were fixated for 10 min with 4% paraformaldehyde in 0.2 M phosphate buffer. Afterward, the cells were washed three times with PBS before the blocking solution (2% bovine serum albumin and 10% goat serum in PBS) was applied for 30 min. Primary antibody solution (anti-KCC2 N1–12; 1:1000 dilution; Neuromab) was added in carrier solution (0.3% Triton X-100, 1% bovine serum albumin, and 1% goat serum in PBS) and incubated for 1 h. After washing three times with PBS, the secondary antibody, which was conjugated to a fluorescent probe (Alexa Flour 488 goat antimouse; 1:1000dilution; Thermo Fisher Scientific), was added to the carrier solution and incubated for 1 h. Again, the cells were washed three times with PBS and completely dried. The dried coverslips were mounted onto glass slides with Mowiol (Roth) and 4′,6-diamidine-2-phenylindole (1:1000 dilution; Roth). Photomicrographs were taken using an Olympus fluorescence microscope (Olympus BX63).

### Determination of the K^+^–Cl^−^ cotransport activity

Transport activity of KCC2 was determined by Cl^−^-dependent uptake of Tl^+^ in HEK-293 cells as described previously (14, 59, 66). To initiate the flux measurement, the medium in the 96-well culture dish was replaced by 80 μl hypotonic preincubation buffer (100 mM *N*-methyl-d-glucamine-chloride, 5 mM Hepes, 5 mM KCl, 2 mM CaCl_2_, 0.8 mM MgSO_4_, 5 mM glucose, pH 7.4; osmolarity: 175 mmol/kg ± 2) with 2 μM FluoZin-2 AM dye (Invitrogen) plus 0.2% (w/v) Pluronic F-127 (Invitrogen) and incubated for 48 min at RT. Afterward, cells were washed three times with 80 μl preincubation buffer and incubated for 15 min with 80 ml preincubation buffer including 0.1 mM ouabain to block the activity of the Na^+^/K^+^ ATPase. Then, the 96-well plate was placed into a fluorometer (Fluoroskan FL), and each well was injected with 40 μl 5× Thallium stimulation buffer (12 mM Tl_2_SO_4_, 100 mM *N*-methyl-d-glucamine, 5 mM Hepes, 2 mM KCl, 2 mM CaCl_2_, 0.8 mM MgSO_4_, 5 mM glucose, pH 7.4). The fluorescence was measured in a kinetic-dependent manner (excitation 485 nm, emission 538 nm, one frame in 6 s in a 200-speriod) across the entire cell population in a single well. By using linear regression of the initial values of the slope of Tl^+^-stimulated fluorescence increase, the transport activity was calculated. The absolute values are normalized by setting the slope of the KCC2^WT^ or KCC2^HA^ construct as 100% and calculating the percentage of the activity of the mutants respectively. Normalization is needed to subtract naturally occurring fluctuations of the fluorescence increase. A comparison of the absolute *versus* normalized values for exemplar mutations can be seen in Figs. S1–S3.

### Statistical analysis

The normalized transport activities of the respective mutant were tested against control samples (*rn*KCC2b^WT^, *mm*KCC2b^HA^, and mock), using a two-sample *t* test after Student’s *t* test for similar variances between samples. In exceptional cases, where the standard deviation differs by more than a factor of 2, Welch's *t* test was used (67). To avoid pseudoreplication, the number of degrees of freedom was deflated according to the size of independent preparations each with three technical replicates. The false discovery rate was controlled, and *p* values were corrected using the Benjamini–Hochberg method (68). The chance of false-positive results (type 1 errors) was reduced by choosing *p* values <0.01. A Tukey honestly significant difference test after Tukey and Kramer was used for multiple comparison for the analyses in supporting information.

## Data availability

All relevant data are available from the corresponding author upon reasonable request.

## Supporting information

This article contains supporting information.

## Conflict of interest

The authors declare that they have no conflicts of interest with the contents of this article.

## Figures and Tables

**Supporting Figure S3:**