# Supplementary material for: Both chloride-binding sites are required for KCC2-mediated transport
Source: J Biol Chem. 2023 Aug 23;299(10):105190. doi: 10.1016/j.jbc.2023.105190 (PMC10518353; doi:10.1016/j.jbc.2023.105190)
Supplement: Supporting Figures S1–S3 [file mmc1.docx]

# **Supplement**

# **Both chloride binding sites are required for KCC2-mediated transport**

**Lisa Becker^1^, Jens Hausmann^2^, Anna-Maria Hartmann^1,3*^**

^1^Division of Neurogenetics, School of Medicine and Health Sciences, Carl von Ossietzky University Oldenburg, 26111 Oldenburg, Germany

^2^ Division of Anatomy, School of Medicine and Health Sciences, Carl von Ossietzky University Oldenburg, 26129 Oldenburg, Germany.

^3^Research Center for Neurosensory Sciences, Carl von Ossietzky University Oldenburg, 26111 Oldenburg, Germany

*Running title: Structural requirements for KCC2 transport activity*

*To whom correspondence should be addressed: Anna-Maria Hartmann, Division of Neurogenetics, Carl von Ossietzky Universität Oldenburg, 26111 Oldenburg, Germany, [anna.maria.hartmann@uol.de](mailto:anna.maria.hartmann@uol.de), Tel. +49-(0)441-798-2937

**Supplementary Figure 1: Comparison of the absolute values of *rn*KCC2b and *mm*KCC2b^HA^**

HEK-293 cells were transiently transfected with KCC2b^WT^ or KCC2^HA^ variants of I^115E^, M^415Q^, and Y^446F^. Tl^+^ flux measurements were performed to determine the transport activity. The graph represents the slope of the fluorescence increase over time. All samples have been measured at the same time, with at least 3 independent measurements including three technical replicates per independent measurement. Statistical analysis for comparison of KCC2b^WT^ against mutants was performed by a Tukey HSD test after Tukey and Kramer with R version 4.2.1 (***: p<0.001; **: p<0.01). KCCs: K^+^- Cl^-^ cotransporter, HSD: honestly significant difference

**Supplementary Figure 2: Comparison of the normalized values of *rn*KCC2b and *mm*KCC2b^HA^**

HEK-293 cells were transiently transfected with KCC2b^WT^ or KCC2^HA^ variants of I^115E^, M^415Q^, and Y^446F^. Tl^+^ flux measurements were performed to determine the transport activity. The graph represents the data that has been normalized according to rnKCC2b. All samples have been measured at the same time, with at least 3 independent measurements including three technical replicates per independent measurement. Statistical analysis for comparison of KCC2b^WT^ against mutants was performed by a Tukey HSD test after Tukey and Kramer with R version 4.2.1 (***: p<0.001; **: p<0.01). KCCs: K^+^- Cl^-^ cotransporter, HSD: honestly significant difference

**Supplementary Figure 3: Immunocytochemistry of *rn*KCC2b and *mm*KCC2b^HA^ variants**

In parallel to the Tl^+^ based flux measurements presented in Suppl. Figs. 1 and 2 an immunocytochemistry analyses were performed to monitor the transfection rate of the KCC2 variants (green) and cell staining by DAPI (blue). Representative immunocytochemical images were used for the biological replicates. The scale bar represents 200 µm.
